# Genome Sequence of *Porphyrobacter dokdonensis* DSW-74^T^, Isolated from Seawater off Dokdo in the East Sea (Sea of Korea)

**DOI:** 10.1128/genomeA.00903-16

**Published:** 2016-09-01

**Authors:** Ju Yeon Song, Juhye Hong, Min-Jung Kwak, Soon-Kyeong Kwon, Jihyun F. Kim

**Affiliations:** a Department of Systems Biology and Division of Life Sciences, Yonsei University, Seodaemun-gu, Seoul, Republic of Korea; b Strategic Initiative for Microbiomes in Agriculture and Food, Yonsei University, Seodaemun-gu, Seoul, Republic of Korea

## Abstract

*Porphyrobacter dokdonensis* strain DSW-74, isolated from seawater off of Dokdo, Republic of Korea, is a member of the family *Erythrobacteraceae*. In this study, the genome sequence of DSW-74 was determined using the Illumina HiSeq 2000 platform and assembled into 11 contigs. Its genome is approximately 3.0 Mb with a G+C content of 64.8%, in which 2,875 protein-coding sequences and 47 RNA genes were predicted.

## GENOME ANNOUNCEMENT

DSW-74 is the type strain of *Porphyrobacter dokdonensis* that was isolated from seawater off Dodko in the East Sea of Korea, and shows optimal growth at slightly halophilic conditions (1). The genus *Porphyrobacter* comprises a group of related Gram-negative, nonsporulating, and aerobic bacteria, and, according to phylogenetic analyses, it belongs to the class *Alphaproteobacteria* (2). Most strains have been isolated from aquatic environments, such as freshwater and marine ecosystems (3). Species of *Porphyrobacter* are photosynthetic: they synthesize bacteriochlorophyll *a* under aerobic conditions and are a red or orange color due to the presence of carotenoid (2). Currently, genome sequences are available for three type strains of *Porphyrobacter* spp.: *P. neustonensis* DSM 9434 (CP016033), *P. cryptus* DSM 12079 (AUHC00000000), and *P. mercurialis* Coronado (4). Additionally, a genome announcement has been published for *Porphyrobacter* sp. AAP82 (5), and several genome sequences have been used for metagenomic studies (6, 7).

Here, we report a summary of the genome sequencing and annotation of *P. dokdonensis* DSW-74. Cells were cultured in marine broth at 37°C for 6 days, and the genomic DNA was isolated according to a method that uses the phenol-chloroform solution (8). The genome sequence was determined using the Illumina HiSeq 2000 platform (NICEM, Republic of Korea). Through whole-genome shogun sequencing, a total of 36,760,806 reads were generated from a 500-bp paired-end library. Along with CLC Genomics Workbench version 5.5.1, sequence trimming and *de novo* assembly were implemented, and scaffolding was carried out with SSPACE (9). The GapCloser software was used to close gaps between the contigs, and manual curation was performed to improve the gaps and to verify the assembly. Automatic annotation was performed using the RAST server (10) and AutoFACT (11). Protein-coding gene sequences were predicted by Glimmer version 3.0 (12), and rRNAs and tRNAs were detected in RAST. The protein-coding genes were aligned to those in the KEGG, COG, UniRef90, Pfam, and GenBank NR databases using BLASTp and RPS-BLAST. Functions of the protein-coding genes were assigned by AutoFACT.

The sequence reads of DSW-74 were assembled into 11 contigs. The assembled genome consists of 2,995,154 bp with a GC content of 64.81%. In the genome, 2,875 protein-coding sequences and 47 RNA genes were predicted, including a single rRNA operon and 44 tRNA genes. A majority of protein-coding genes (82.78%) were assigned with a putative function, including a gene cluster involved in photosynthesis, while remaining genes were annotated as hypothetical. As the first genome available for *P. dokdonensis*, this work expands the repertoire of genomic information for the genus *Porphyrobacter* and will be useful in several studies, including comparisons between genomes and characterization of ecological functions for photosynthetic bacteria.

### Accession number(s).

The draft genome sequence of *Porphyrobacter dokdonensis* DSW-74 has been deposited in GenBank under the accession number LZYB00000000.
